# Structure-Based Virtual Screening of Furan-1,3,4-Oxadiazole Tethered *N*-phenylacetamide Derivatives as Novel Class of hTYR and hTYRP1 Inhibitors

**DOI:** 10.3390/ph16030344

**Published:** 2023-02-23

**Authors:** Ali Irfan, Shah Faisal, Sajjad Ahmad, Sami A. Al-Hussain, Sadia Javed, Ameer Fawad Zahoor, Bushra Parveen, Magdi E. A. Zaki

**Affiliations:** 1 Department of Chemistry, Government College University Faisalabad, Faisalabad 38000, Pakistan; 2 Department of Chemistry, Islamia College University Peshawar, Peshawar 25120, Pakistan; 3 Department of Health and Biological Sciences, Abasyn University, Peshawar 25000, Pakistan; 4 Department of Chemistry, College of Science, Imam Mohammad Ibn Saud Islamic University (IMSIU), Riyadh 11623, Saudi Arabia; 5 Department of Biochemistry, Government College University Faisalabad, Faisalabad 38000, Pakistan

**Keywords:** furan-1,3,4-oxadiazole, hTYR, hTYRP1, melanogenesis, molecular docking, MD simulations

## Abstract

Human tyrosinase (hTYR) is a key and rate-limiting enzyme along with human tyrosinase-related protein-1 (hTYRP1), which are among the most prominent targets of inhibiting hyper pigmentation and melanoma skin cancer. In the current in-silico computer-aided drug design (CADD) study, the structure-based screening of sixteen furan-1,3,4-oxadiazole tethered *N*-phenylacetamide structural motifs **BF1**–**BF16** was carried out to assess their potential as hTYR and hTYRP1 inhibitors. The results revealed that the structural motifs **BF1**–**BF16** showed higher binding affinities towards hTYR and hTYRP1 than the standard inhibitor kojic acid. The most bioactive lead furan-1,3,4-oxadiazoles **BF4** and **BF5** displayed stronger binding in affinities (−11.50 kcal/mol and −13.30 kcal/mol) than the standard drug kojic acid against hTYRP1 and hTYR enzymes, respectively. These were further confirmed by MM-GBSA and MM-PBSA binding energy computations. The stability studies involving the molecular dynamics simulations also provided stability insights into the binding of these compounds with the target enzymes, wherein it was found that they remain stable in the active sites during the 100 ns virtual simulation time. Moreover, the ADMET, as well as the medicinal properties of these novel furan-1,3,4-oxadiazole tethered *N*-phenylacetamide structural hybrids, also showed a good prospect. The excellent in-silico profiling of furan-1,3,4--oxadiazole structural motifs **BF4** and **BF5** provide a hypothetical gateway to use these compounds as potential hTYRP1 and hTYR inhibitors against melanogenesis.

## 1. Introduction

Skin cancer is the one of the most common cancers which adversely affect humans. The main types of skin cancer include melanoma, basal cell carcinoma and squamous. Among them, melanoma is far less frequent than the other types. Melanoma cancer has a higher propensity to spread to other body areas by invading adjacent tissue [1]. Melanoma is the skin cancer that leads to the majority of fatalities, with an average increase of roughly one million new cases per year. Skin cancer has developed into the most prevalent malignant ailment, accounting for 4.5% of all new cancer cases, and it continues to be a fatal cancer that causes significant socioeconomic challenges [2,3,4]. When melanomas are in an advanced stage, they are required to be treated surgically and with adjuvant systemic therapies [5]. As with other malignancies, radiation can be used alone or in combination with surgery to treat melanoma. However, radiotherapy has a limited function in the treatment of melanoma since it is radio-resistant in comparison to other malignancies [6]. Thus, the development of drugs that specifically target cell-signaling pathways involved in this malignancy holds promise for the treatment of melanomas [7,8]. The enzyme human tyrosinase (hTYR) and human tyrosinase-related protein-1 (hTYRP1) are involved in the biosynthetic processes that produce the pigment melanin in the melanocytes. The hTYR and hTYRP1 have been shown to be sensitive melanoma biomarkers and these are also overexpressed during carcinogenesis [9,10,11]. Additionally, the melanin biosynthesis pathway produces powerful immunosuppressive intermediate species such as L-DOPA and other reactive quinines which further aggravate melanomas by negating the anti-melanoma actions of the immunotherapeutic medications that target these malignancies [12,13,14,15].

All the observations and factors link the elevated melanogenesis as a cause of the lethality of skin melanomas due to the increased activity and overexpression of hTYR and hTYRP1 [13,16]. Therefore, targeting the inhibition of crucial enzymes hTYR and hTYRP1 with suitable inhibitors prevents the formation of melanomas and may help in treating these cancers [13,17,18]. To date, several approaches of in-vivo studies targeting the melanin biosynthetic pathway for treating melanomas have been reported [19]. hTYR, hTYRP1 and the other related enzymes of the melanin biosynthetic pathway are possible molecular targets in the treatment of melanoma and other melanogenesis-related disorders due to their overexpression, which occurs during carcinogenesis primarily in the melanocytes [13,20,21].

**Figure 1:**
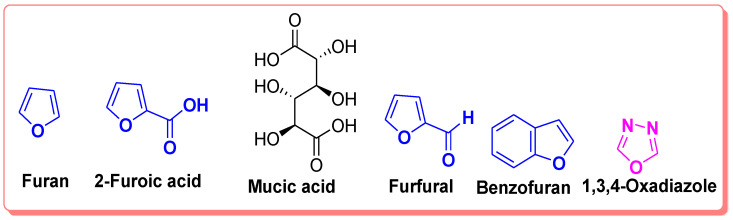
Structures of furan, mucic acid, 1,3,4-oxadiazole and furan-related compounds.

The plethora of literature cited in previous studies revealed that numerous furans containing molecules, benzofurans (Figure 1), 1,3,4-oxadiazoles (Figure 1), furan-1,3,4-oxadiazoles and other furan moiety carrying scaffolds are effective inhibitors of mushroom and human tyrosinases. The origin of furan chemistry has been outlined by Partington and the furan first derivative pyromucic acid (furan-2-carboxylic acid) or simply 2-furoic acid (Figure 1) was obtained via dry distillation of mucic acid (Figure 1). In 1831, Johann Wolfgang Döbereiner reported another important derivative of furan called furfural (Figure 1) (furan-2-carbaldehyde), which was further characterized by John Stenhouse. Heinrich Limpricht isolated furan for the first time in 1870 from pine wood, called tetraphenol due to the presence of four carbon atoms and strong resemblance to phenol in many reactions, e.g., with bromine [22,23,24,25].

**Figure 2:**
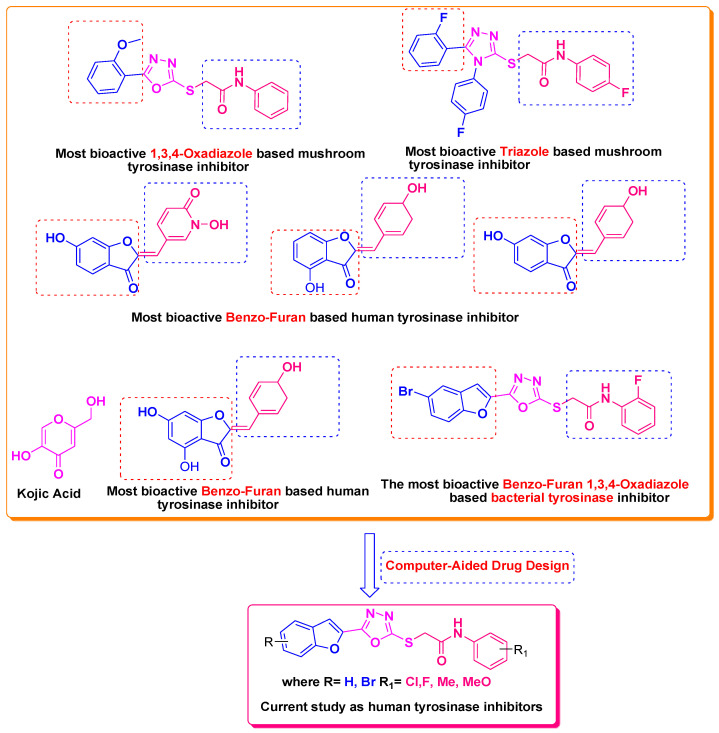
Bioactive tyrosinase inhibitors.

Furan, benzofuran and other nitrogen-oxygen heterocyclic structural motifs had demonstrated excellent medicinal and pharmacological profiles, and exhibited a wide spectrum of biological activities such as antibacterial, anti-fungal, anti-diabetic, anti-acetylcholine, anti-viral, anti-inflammatory, anti-parasitic, fluorescent sensor for analgesic, anti-HepG-2, anti-oxidative, bone anabolic agent and as bacterial tyrosinase inhibitors; they are also part of many natural and synthetic clinical drugs. In various research models, furan scaffolds have shown potent anti-tyrosinase actions which imply that these compounds are effective inhibitors of the melanin biosynthesis in the melanocytes and can be used in treating skin melanomas and other melanogenesis-related disorders, as depicted in Figure 2 [26,27,28,29,30,31,32,33,34,35,36,37,38].

Our previous work reported the evaluation of benzofuran-1,3,4-oxadiazole tethered *N*-phenylacetamides as bacterial tyrosinase inhibitors and showed strong repressive activities of benzofuran compounds, which encouraged us to apply computer-aided drug discovery (CADD) approaches to assess the therapeutic potential of sixteen synthesized furan-1,3,4-oxadiazole tethered *N*-phenylacetamide structural hybrids, **BF1–BF16 [38,39]**, with different substituents to target the crucial enzymes hTYR and hTYRP1 of the melanin biosynthetic pathway.

**Figure 3:**
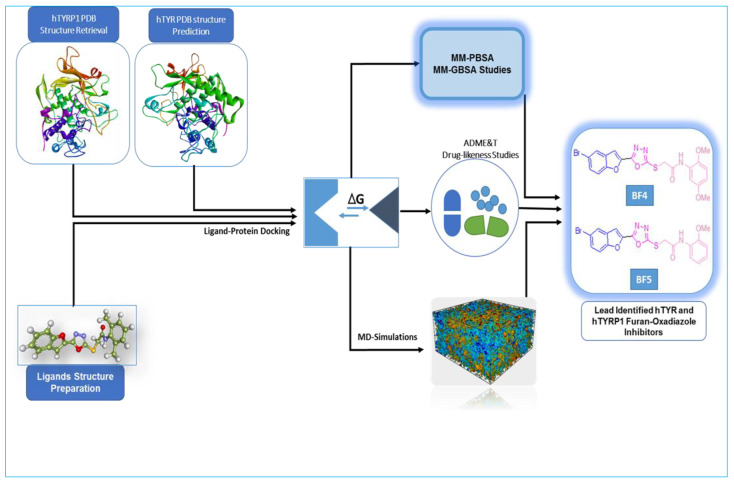
In silico work flow for discovery of novel hTYR and hTYRP1 inhibitors via CADD.

The therapeutic potential of synthesized furan–oxadiazole scaffolds **BF1–BF16** was assessed utilizing a computer-aided drug design (CADD) workflow as displayed in Figure 3.

## 2. Results and Discussion

### 2.1. Computational Investigations of BF1–BF16 against hTYR and hTYRP1

**Figure 4:**
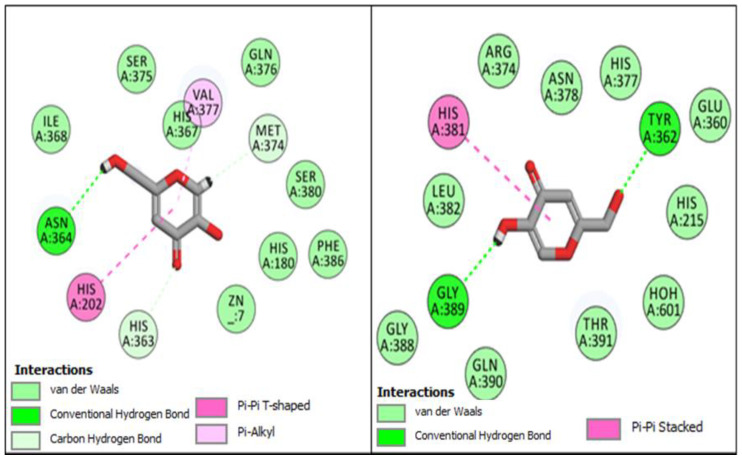
Conformational poses of kojic acid with hTYR (**Left panel**) and hTYRP1 (**Right panel**).

Utilizing the in silico molecular docking approach, we evaluated furan-1,3,4-oxadiazoles synthesized compounds **BF1–BF16** using the molecular operating environment (MOE) against the hTYR and hTYRP1 of the melanin synthesis pathway and compared these results with the standard tyrosinase inhibitor drug kojic acid, which has been shown to repress these two enzymes (hTYR and hTYRP1) in various studies. The results of these investigations revealed that the anti-hTYR and hTYRP1 repressive agent kojic acid binds to the active site of the hTYR enzyme with a binding affinity score of −6.62 Kcal/mol, and it binds to the hTYRP1 with a binding affinity score of –8.90 Kcal/mol. Conformation analysis of the kojic acid inhibitor inside the active site pocket of hTYR revealed that it engaged multiple amino acids residues (ASN364, HIS367,and MET374) and made conventional-type and carbon–hydrogen-type hydrogen bonds with it; along with several other molecular interactions of Pi–Pi T-shaped and Pi–Alkyl-type interactions with HIS202 and VAL377 were also observed in the hTYR and kojic acid protein–ligand complex. Binding analysis of kojic acid with the hTYRP1 enzyme showed that it made conventional hydrogen bonds with the TYR362 and GLY389 active site residues and a Pi–Pi stacked interaction with the HIS381. The conformational poses of kojic acid with both enzymes are presented in Figure 4.

**Figure 5:**
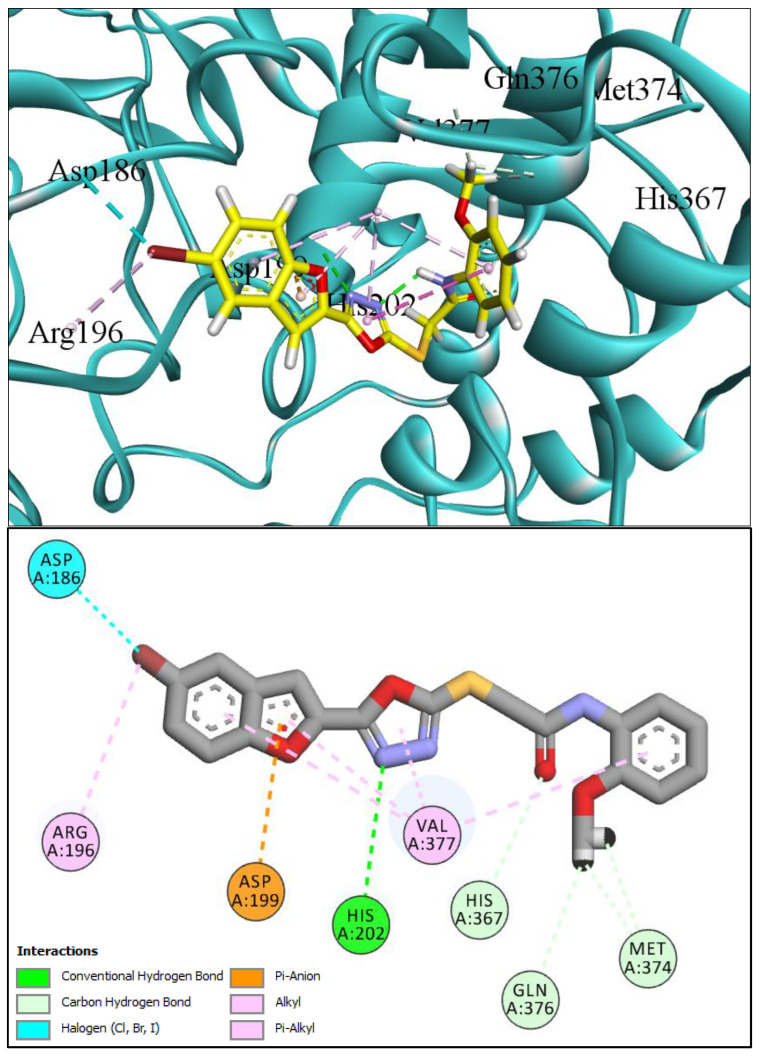
Three-dimensional conformational pose of BF5 with hTyrosinase (**Upper panel**) and two-dimensional pose (**Lower panel**).

In comparison with the kojic acid inhibitor, some of the synthesized benzofuran compounds showed robust interactions and higher binding affinities with the hTYR and hTYRP1 enzymes of the melanin synthesis pathway. Out of the sixteen compounds, **BF1–BF16**, three compounds, **BF5**, **BF7**, and **BF15,** showed stronger affinities against the hTYR, while the other three, **BF4**, **BF5**, and **BF7**, showed stronger affinities against the hTYRP1 compared to the standard drug kojic acid. Analysis of the conformational pose and binding affinity of **BF5** showed that it binds to the hTYR active site with a binding affinity score of −13.30 Kcal/mol and forms multiple molecular interactions with the hTYR enzyme receptor residues. Hydrogen bonds of conventional and carbon–hydrogen types were noted between the benzofuran ring of **BF5** and HIS202, HIS367, GLN376, MET374 residues. Moreover, other interactions, such as Pi–Anion, Alkyl, and Pi–Alkyl interactions (with the VAL377), were also present between the 1,3,4-oxadiazole and benzofuran rings of this compound; in addition, several of the hTyrosinase receptor residues (ASP186 and ARG196) also made halogen molecular interactions with the bromine present on the benzofuran ring of this compound. These are diagrammatically presented in Figure 5.

**Figure 6:**
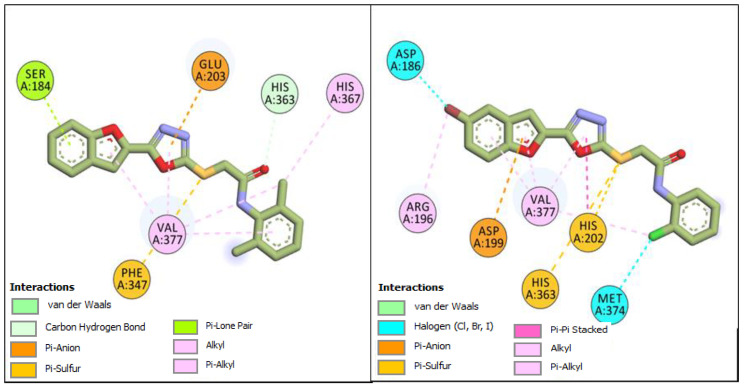
Two-dimensional conformational pose of **BF7** with hTyrosinase (**Right panel**) and two-dimensional conformational pose of **BF15** with hTyrosinase (**Left panel**).

The two other compounds, **BF7** and **BF15,** were also able to bind to the hTyrosinase with higher binding affinities (−11.19 Kcal/mol and −11.88 Kcal/mol, respectively) compared to the kojic acid, which was able to bind to the hTYR with a binding affinity of −6.62 Kcal/mol. These two compounds were also able to engage multiple active site residues of hTYR via a different type of molecular interactions. Overall, the **BF7** compound showed a similar kind of binding conformation and interactions with the hTyrosinase enzyme; however, the sulfur atom of **BF7** made two more Pi–sulfur interactions with the HIS363, and HIS202 and the chlorine atom present on the phenyl ring of the **BF7** made two halogen interactions with the VAL377 and MET374 active site residues. Similarly, **BF15** also showed robust binding with the hTyrosinase by engaging the HIS363 with a carbon–hydrogen-type hydrogen bond, while PHE347 made a molecular contact via a Pi–sulfur interaction with the sulfur atom of **BF15**. Several other types of hydrophobic interactions were also observed between the benzofuran, 1,3,4- oxadiazole, and the phenyl ring of **BF15,** and they can be seen in Figure 6.

**Figure 7:**
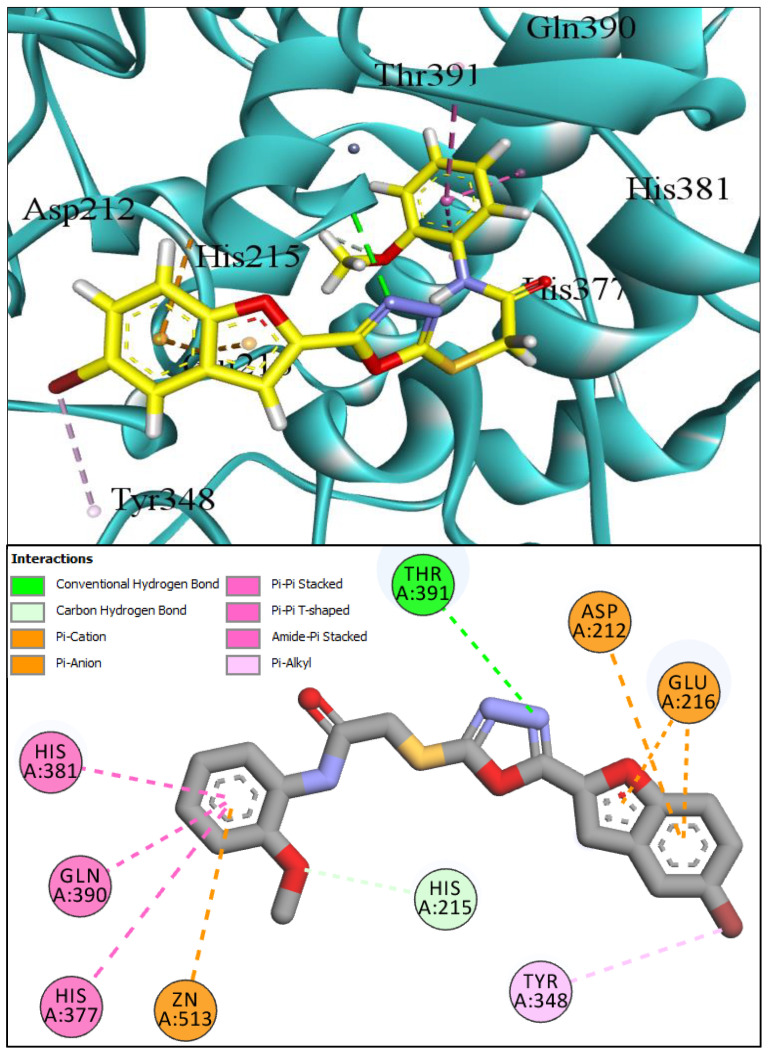
Three-dimensional conformational pose of **BF5** with human tyrosinase-related protein-1 (hTYRP1) (**Upper panel**) and its two-dimensional pose (**Lower panel**).

The molecular docking investigations against the hTYRP1 enzyme also revealed that these newly synthesized compounds show superior binding and interactions with this important enzyme. The standard inhibitor kojic acid, as discussed in the previous paragraphs, binds to the hTYRP1 with a binding affinity score of –8.90 Kcal/mol; compared to kojic acid, some of these new furan-1,3,4-oxadiazoles (**BF4**, **BF5**, and **BF7**) have shown good binding affinities towards the hTYRP1 enzyme. The furan-1,3,4-oxadiazoles **BF4, BF5** and **BF7** were able to bind to the hTYRP1 with binding affinities of −11.50 Kcal/mol, −11.55 Kcal/mol, and −11.29 Kcal/mol, respectively. The conformational pose analysis of **BF4** in the active site of the hTYRP1 enzyme showed that it binds with the active pocket residues via different types of molecular interactions. The acetamide group, as well as the oxygen atom of the 1,3,4-oxadizole ring present in this compound, made conventional hydrogen bonds with the ARG374 and THR391 amino acids of the hTYRP1, and a carbon–hydrogen-type H-bond between the –OCH_3_ and the SER394 amino acid was also present in this ligand–protein complex. The 1,3,4-oxadiazole ring of **BF4** also made another Pi–anion interaction with the GLU216 of the hTYRP1 active site, while the bromine atom on the benzofuran ring and the phenyl ring of BF-4 made Alkyl and Pi–Alkyl interactions with LEU293 and LYS198 residues of hTYRP1. **BF5,** which showed the highest binding affinity (−11.55 Kcal/mol) towards the hTYRP1 active site, also showed robust interactions of different types with this target enzyme. The phenyl ring of **BF-5** made a total of four interactions with the HIS381, GLN390, and HIS377 of Pi–Pi stacked, Pi-Pi T-shaped and amide–Pi stacked type along with a direct interaction with the zinc ion present in the active site of the hTYRP1. The 1,3,4-oxadiazole ring engaged the THR391 residue while the benzofuran ring made three Pi–anion interactions with the ASP212 and GLU216; however, the –OCH_3_ present on the phenyl ring made a single carbon–hydrogen-type H-bond with the HIS215 of the hTYRP1 enzyme active site. Figure 7 shows the three and two-dimensional conformations of **BF5** inside the hTYRP1 active site.

**Figure 8:**
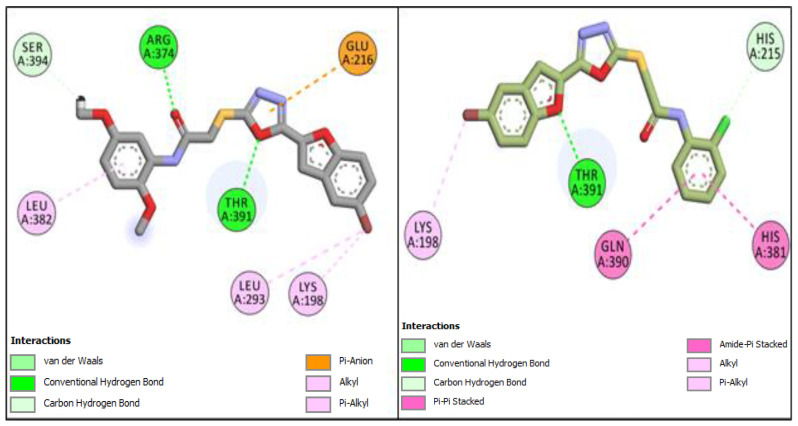
Two-dimensional conformational pose of **BF4** with hTYRP1 (**Left panel**) and two-dimensional conformational pose of **BF7** with hTYRP1 (**Right panel**).

The furan-1,3,4-oxadiazole **BF7** compound with a binding affinity of −11.29 Kcal/mol with the hTYRP1 also exhibited several different types of interactions with the LYS198, HIS215, HIS381, GLN390, and THR391 active sites. Figure 8 shows the two-dimensional poses of **BF4** and **BF7** with the hTYRP1 enzyme.

The binding affinities of the biologically active furan-1,3,4-oxadiazole compounds against hTYR and hTYRP1 are shown in Table 1.

### 2.2. ADMET and Drug-Likeness Predictive Studies

The pharmacokinetics, or, in short, the ADMET studies along with the drug-likeness in silico investigations, showed that the furan-1,3,4-oxadiazole compounds have significantly good GI-Tract absorption values and they were listed as HIA+. These compounds were listed to have acceptable lipophilic (iLogP) properties and also had good water solubility (LogS-ESOL) values. They were non-inhibitors of the P-gp protein and non-substrates of the CYP450-3A4 enzyme. They were non-inhibitors of renal (OCTs) and were found to be non-AMES toxic. Along with these good ADMET properties, these compounds also showed good medicinal chemistry profiles and accepted the Lipinski rule, Pfizer rule and Golden triangle rule. Table 2 has the different pharmacokinetic properties listed, while Table 3 contains the medicinal chemistry profiles of the best lead compounds identified in this investigation.

### 2.3. Molecular Dynamics Stability Analysis of the Ligand-Protein Complexes

**Figure 9:**
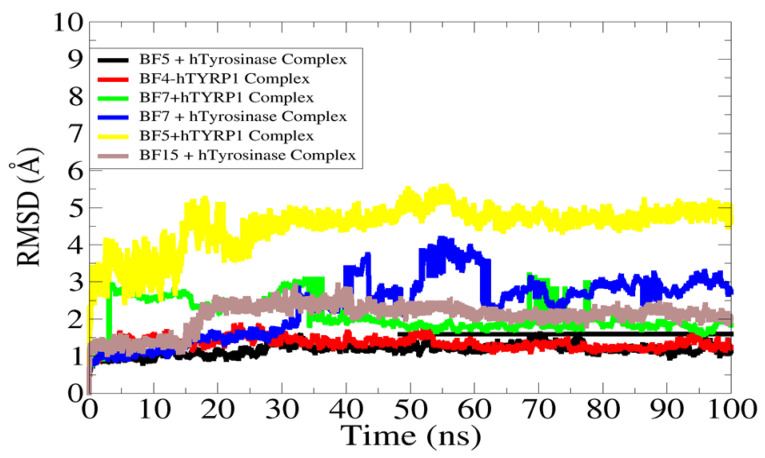
RMSD analysis of complexes to decipher their dynamic stability.

All atoms’ molecular dynamic simulations were conducted for complexes in order to understand and interpret intermolecular dynamics and the stability of docked molecules with the receptor enzymes. As a bio-molecule function in dynamics inside the cells, it is important to evaluate the dynamic behavior rather than focusing on static nature. The trajectories of simulations were generated using the root mean square deviation (RMSD) statistical parameter. RMSD measure the all-atom carbon alpha mean deviation with respect to initial reference position versus time. Higher RMSD describes more structure deviations, while lower RMSD points to small structure changes. The RMSD plot for each complex is provided in Figure 9. The **BF4**-hTYRP1 (mean RMSD of 0.98 Å), **BF15** + hTYR (mean RMSD of 1.62 Å), **BF5** + hTYR (mean RMSD of 0.88 Å) and **BF7** + hTYRP1 (mean RMSD of 1.86 Å) were the most stable complexes that showed little structure deviations in the simulated time. The mean RMSD of these complexes was 1.2 Å. This stable nature of complexes enables the receptors’ 3D structure to remain confined and fixed in the presence of compounds during simulation time. The **BF5** + hTYRP1, on the other hand, reported the highest RMSD, which touches almost five angstroms. It is very clear in the analysis that all complexes, after initial small deviation, attained considerable structure stability. The data showed that the compounds are stably docked inside the active pocket of enzymes, and the binding conformation, except for a few initial small adaptations, remained stable throughout the length of simulation time.

**Figure 10:**
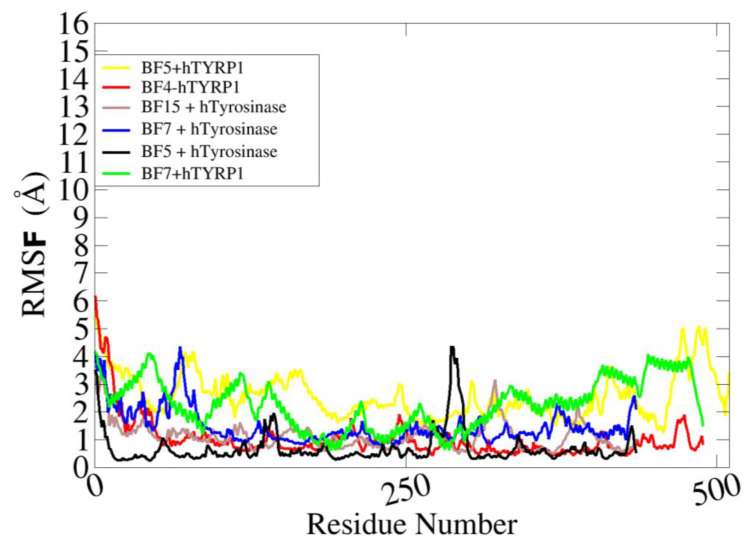
Residue level fluctuation of complexes.

To obtain a further understanding of the residue level flexibility, root mean square fluctuation (RMSF) analysis was conducted. The mean RMSF of BF5 + hTyrosinase, BF7 + hTyrosinase, BF15 + hTyrosinase, BF4-hTYRP1, BF5 + hTYRP1, and BF7 + hTYRP1 is 1.1 Å, 2.2 Å, 0.8 Å, 1.8 Å,2.7 Å, and 2.9 Å, respectively. All the systems reported stable fluctuations at the residue level with major deviations seen at loops. The RMSF of the complexes is shown in Figure 10.

### 2.4. MM-GBSA/MM-PBSA Binding Free Energy Analysis of Complexes

The binding free energies calculation using MMGBSA and MMPBSA is a significant approach to revalidate the docking results, as they are more reliable and use modest computational power. The order of complexes based on stable net-binding energy is in the following order: **BF4**-hTYRP1 > **BF4**-hTYRP1 > **BF7** + hTYRP1 > **BF5** + hTYR > **BF7** + hTYR > **BF5** + hTYRP1. Generally, the gas phase energy in all complexes was found to dominate the chemical interaction network between the docked molecules and enzymes’ active pocket residues. Decomposing the gas phase energy, the van der Waals energy component was the most dominating, ranging from −55.01 kcal/mol for **BF4**-hTYRP1 and −48.62 kcal/mol for **BF5** + hTYRP1. In addition to that, electrostatic energy played a major role in intermolecular complex formation. The highest contribution was seen in the case of −31.74 kcal/mol for **BF4**-hTYRP1 and the lowest contribution was seen in the case of −22.17 kcal/mol for **BF5** + hTYRP1. The non-favorable contribution was reported from solvation energy, which was highest at 28.99 kcal/mol in **BF5** + hTYR and lowest at 23.59 kcal/mol in **BF7** + hTYRP1. The net-binding energy along with each energy parameter value (kcal/mol) is shown in Table 4.

## 3. Material and Methods

### 3.1. Structures of Synthsized Furan-1,3,4-Oxadiazoles BF1–BF16

The structures of furan-1,3,4-oxadiazole structural motifs, which were synthesized by Irfan, A et al. [38,39], are shown in Table 5.

### 3.2. In Silico Biological Evaluation of Furan-1,3,4-Oxadiazoles BF1–BF16

#### 3.2.1. Molecular Docking, ADME&T, Drug-Likeness, and Protein Homology Modeling Studies

The PDB structure of the target enzyme hTYR was predicted with homology modeling via the Swiss-Model prediction server using the fasta sequence of human tyrosinase with Uniprot ID = P14679 (because its resolved crystal structure is not available yet), while the structure of the hTYRP1 PDB ID-5M8M was accessed from the RCSB website for the computational investigations. The molecular docking investigations were performed via the Molecular Operating Environment (MOE) (Version-2009.10). Before docking, the protein structure of the hTYR and hTYRP1 enzyme was prepared for docking studies using the Biovia DS software. The structures of ligands **BF1–BF16** were prepared using the ChemDraw Professional. The (.mol) format structure of these ligands was imported into MOE, where the partial charges were added to them along with energy minimization of these compounds, which was performed using the MMFF94x –ff. In MOE, the protein structures were loaded and 3D-protonated; after that, its site-finder function was utilized for the active site identification. The triangle matcher technique and the London-dG scoring functions were used for the binding affinity estimations of these compounds against the target enzymes by the DOCK module of the MOE Software. Furthermore, the BIOVIA DS (Version-2017) software was utilized for the interaction analysis and visualization of the ligand–protein complexes [40,41,42,43,44,45,46,47,48,49]. The ADMET and drug-likeness investigation were carried out using the Swissadme and ADMETlab (Version 2.0) online servers, while the admetSAR (Version 1.0 and 2.0) online servers were utilized for the toxicity investigations of these compounds [50,51,52,53]. 

#### 3.2.2. Molecular Dynamic Simulation Studies

For all docked complexes, the computer-based molecular dynamic simulations were performed employing AMBER (Version-20) simulation software. This analysis was important to conduct in order to understand protein–ligand’s stability and dynamics in simulated time scale. In order to define parameters for the proteins and compounds, AMBER FF14SBand GAFF force fields were used, respectively. Charge assignment was carried out using the AMBER antechamber program. The placement of complexes into TIP3 simulation box was then accomplished by setting the distance between the molecules and box edge as 12 Å. Addition of counter ions was performed to obtain neutral systems. Long-range electrostatic interactions were evaluated using the Ewald summation method. The SHAKE algorithm was applied to constrain bounded hydrogen atoms, while to control temperature and pressure, Langevin and Berendson’s barostats were run, respectively [54,55,56,57,58,59,60].

Prior to production, the complexes were minimized for energy in two stages; first by fast steepest descent for 10,000 steps followed by slow conjugate gradient for 15,000 steps. The complexes were then heated to 300 K. The equilibration of complexes was achieved using NPT and NVE ensembles with a collision frequency of 2. The production run was performed for 100 ns and the generated trajectories were evaluated through the CPPTRAJ tool [61]. The statistical plots were produced using XMGRACE (Version-5.1).

#### 3.2.3. Estimation of Binding Energies and Interactions (MM-GBSA and MM-PBSA)

Estimation of binding interactions was carried out using the molecular mechanics Poisson–Boltzmann surface area (MM-PBSA) technique of AMBER (Version-20). This involves the calculation of continuum electrostatics, molecular mechanics, and solvent-accessible surface area [62,63]. Equation (1) was used for this calculation to describe the difference between the energy of the complex, receptor and ligand, as shown below (Equation (1)) [64,65].

Δ*G*_bind_ = *G*_complex_ − *G*_protein_ − *G*_ligand_ = Δ*E*_MM_ + Δ*G*_sol_ − TΔS (1)

The equation was performed on 1000 simulation frames and the net-binding energies were estimated as a sum of gas phase and solvation energies.

## 4. Conclusions

In conclusion, we have evaluated sixteen furan-1,3,4-oxadiazole tethered *N*-phenylacetamides, **BF1–BF16**, which were evaluated via in silico analysis for melanogenesis. We have screened out the most bioactive *N*-phenylacetamide-based furan-1,3,4-oxadiazole scaffolds, **BF4** and **BF5**, against hTYRP1 and hTYR, respectively. Among these promising molecules, 2,5-dimethoxy containing furan-1,3,4-oxadiazole, **BF4**, displayed stronger binding in affinity (−11.50 kcal/mol) against hTYRP1, and 2-methoxy containing furan-1,3,4-oxadiazole, **BF5**, exhibited the best binding affinity (−13.30 kcal/mol) against hTYR compared to the binding in affinities of its standard inhibitor drug kojic acid. Furthermore, the MM-GBSA/MM-PBSA binding energy analysis also showed that these two compounds, **BF4** and **BF5**, bind strongly with the target enzymes hTYRP1 and hTYR. The molecular dynamics simulations stability analysis of the simulation trajectories of these complexes after the 100 ns simulation time provided insights into the stable bindings of these *N*-phenylacetamide-based furan-1,3,4-oxadiazole compounds (**BF4** and **BF5)** within the hTYRP1 and hTYR enzymes active sites. The furan-1,3,4-oxadiazole tethered *N*-phenylacetamide structural hybrids **BF4** and **BF5** also exhibited good ADMET and drug-likeness properties, which suggests the suitability of these compounds as potent inhibitory drug candidates against hTYRP1 and hTYR.

## Figures and Tables

**Table 1:** Binding affinities of the best binding compounds with the hTyrosinase and hTYRP1 enzymes.

Compound	Binding Affinity in (Kcal/mol) with hTYRP1	Binding Affinity in (Kcal/mol) with hTYR
**BF4**	−11.50 Kcal/mol	--
**BF5**	−11.55 Kcal/mol	−13.30 Kcal/mol
**BF7**	−11.29 Kcal/mol	−11.19 Kcal/mol
**BF15**	--	−11.88 Kcal/mol
**Kojic acid** **(Standard)**	−8.90 Kcal/mol	−6.62 Kcal/mol

**Table 2:** ADMET profile of **BF4**, **BF5**, **BF7** and **BF15** furan-1,3,4-oxadiazole compounds.

Compounds	HIA+ Values	Lipophilicity (iLogP)	CYP450 3A4 Inhibitor/ Substrate	Water Solubility	P-gp Substrate	Carcinogenicity	Renal (OCTs)
**BF4**	1.0	3.99	Substrate	Moderately soluble	No	None	Non Inhibitor
**BF5**	1.0	3.75	Substrate	Moderately soluble	No	None	Non Inhibitor
**BF7**	1.0	3.66	Substrate	Poorly soluble	No	None	Non Inhibitor
**BF15**	1.0	3.57	Substrate	Moderately soluble	No	None	Non Inhibitor

**Table 3:** Drug-likeness profile of **BF4**, **BF5**, **BF7** and **BF15** furan-1,3,4-oxadiazole compounds.

Compounds	Bioavailability Score	PAINS Alerts	Brenk Alerts	Lipinski Rule	Pfizer Rule	Golden Triangle Rule	TPSA
**BF4**	0.55	None	None	Accepted	Accepted	Accepted	124.92 Å²
**BF5**	0.55	None	None	Accepted	Accepted	Accepted	115.69 Å²
**BF7**	0.55	None	None	Accepted	Accepted	Accepted	106.46 Å²
**BF15**	0.55	None	None	Accepted	Accepted	Accepted	106.46 Å²

**Table 4:** Different energy contribution to net binding energy of complexes.

Energy Parameter	BF5 + hTYR (SD)	BF7 + hTYR (SD)	BF15 + hTYR (SD)	BF4-hTYRP1 (SD)	BF5 + hTYRP1 (SD)	BF7 + hTYRP1 (SD)
**MM-GBSA**						
**Van der Waals**	−52.20(4.25)	−50.28 (3.68)	−52.62 (5.72)	−55.01 (5.07)	−48.62 (4.68)	−49.55 (5.21)
**Electrostatic**	−23.89(3.65)	−24.01 (3.56)	−28.21 (2.51)	−31.74 (4.68)	−22.17 (3.66)	−26.30 (4.22)
**Delta G gas**	−76.09(6.84)	−74.29 (5.38)	−80.83 (8.60)	−86.75 (6.81)	−70.79 (7.25)	−75.85 (6.38)
**Delta G solv**	28.99 (2.38)	27.68 (7.48)	24.86 (3.84)	25.08 (4.29)	28.64 (4.21)	23.59 (3.56)
**Delta Total**	−47.1 (5.52)	−46.61 (6.87)	−55.97 (5.69)	−61.67 (6.31)	−42.15 (6.11)	−52.26 (3.98)
**MM-PBSA**						
**Van der Waals**	−52.20(4.25)	−50.28 (3.68)	−52.62 (5.72)	−55.01 (5.07)	−48.62 (4.68)	−49.55 (5.21)
**Electrostatic**	−23.89(4.25)	−24.01 (3.56)	−28.21 (2.51)	−31.74 (4.68)	−22.17 (3.66)	−26.30 (4.22)
**Delta G gas**	−76.09(6.84)	−74.29 (5.38)	−80.83 (8.60)	−86.75 (6.81)	−70.79 (7.25)	−75.85 (6.38)
**Delta G solv**	25.60 (1.68)	25.07 (2.58)	28.61 (3.64)	24.01 (3.29)	27.64 (4.62)	24.50 (3.33)
**Delta Total**	−50.49(4.68)	−49.22 (5.28)	−52.22 (4.22)	−62.74 (2.67)	−43.15 (3.08)	−51.35 (2.67)

**Table 5:** Structures of synthetic furan-1,3,4-oxadiazoles **BF1–BF16**.

Compounds	Structures of Furan-1,3,4-Oxadiazssoles
**BF1**	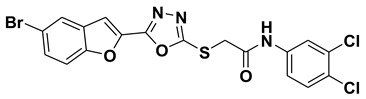
**BF2**	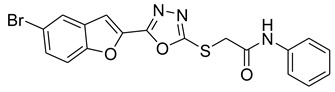
**BF3**	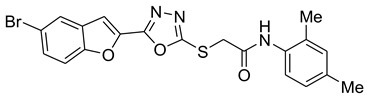
**BF4**	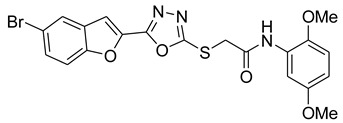
**BF5**	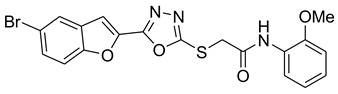
**BF6**	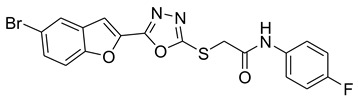
**BF7**	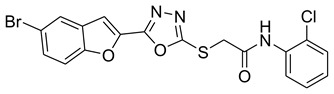
**BF8**	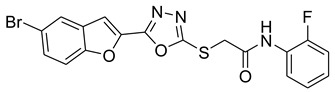
**BF9**	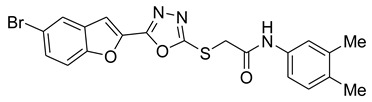
**BF10**	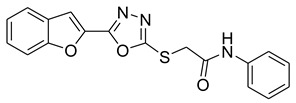
**BF11**	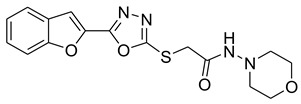
**BF12**	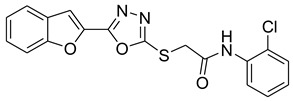
**BF13**	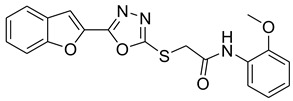
**BF14**	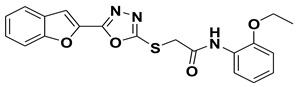
**BF15**	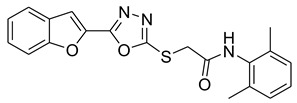
**BF16**	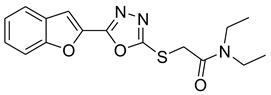

## Data Availability

Data is contained within the article.
